# Preparation and Characterization of Pulp and Paper Mill Sludge-Activated Biochars Using Alkaline Activation: A Box–Behnken Design Approach

**DOI:** 10.1021/acsomega.2c04290

**Published:** 2022-09-02

**Authors:** Glaydson Simões dos Reis, Davide Bergna, Sari Tuomikoski, Alejandro Grimm, Eder Claudio Lima, Mikael Thyrel, Nils Skoglund, Ulla Lassi, Sylvia H. Larsson

**Affiliations:** † Department of Forest Biomaterials and Technology, Swedish University of Agricultural Sciences, Biomass Technology Centre, SE-901 83 Umeå, Sweden; ‡ Research Unit of Sustainable Chemistry, University of Oulu, PO Box 4300, FI-90014 Oulu, Finland; § Unit of Applied Chemistry, University of Jyvaskyla, Kokkola University Consortium Chydenius, Talonpojankatu 2B, FI-67100 Kokkola, Finland; ∥ Institute of Chemistry, Federal University of Rio Grande do Sul (UFRGS), Av. Bento Gonçalves 9500, Porto Alegre 91501-970, RS, Brazil; ⊥ Thermochemical Energy Conversion Laboratory, Department of Applied Physics and Electronics, Umeå University, SE-901 87 Umeå, Sweden

## Abstract

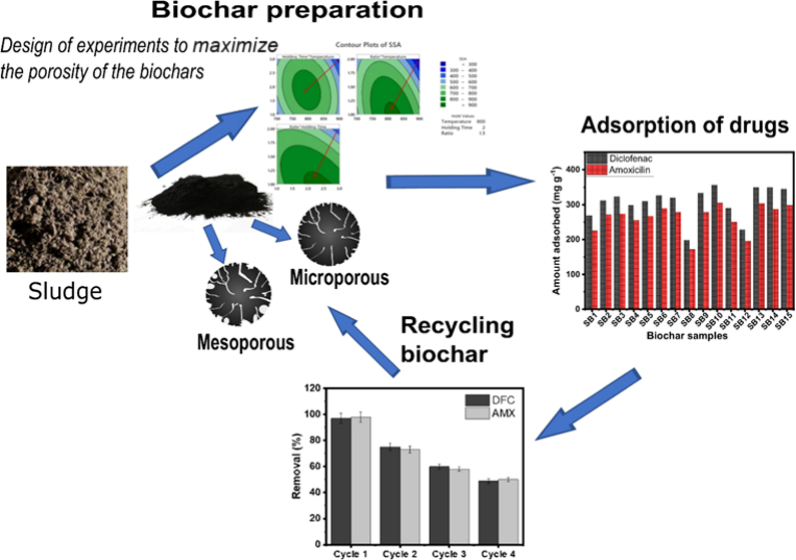

This study utilized pulp and paper mill sludge as a carbon source to produce activated biochar adsorbents. The response surface methodology (RSM) application for predicting and optimizing the activated biochar preparation conditions was investigated. Biochars were prepared based on a Box–Behnken design (BBD) approach with three independent factors (i.e., pyrolysis temperature, holding time, and KOH:biomass ratio), and the responses evaluated were specific surface area (SSA), micropore area (*S*_micro_), and mesopore area (*S*_meso_). According to the RSM and BBD analysis, a pyrolysis temperature of 800 °C for 3 h of holding and an impregnation ratio of 1:1 (biomass:KOH) are the optimum conditions for obtaining the highest SSA (885 m^2^ g^–1^). Maximized *S*_micro_ was reached at 800 °C, 1 h and the ratio of 1:1, and for maximizing *S*_meso_ (569.16 m^2^ g^–1^), 800 °C, 2 h and ratio 1:1.5 (445–473 m^2^ g^–1^) were employed. The biochars presented different micro- and mesoporosity characteristics depending on pyrolysis conditions. Elemental analysis showed that biochars exhibited high carbon and oxygen content. Raman analysis indicated that all biochars had disordered carbon structures with structural defects, which can boost their properties, e.g., by improving their adsorption performances. The hydrophobicity–hydrophilicity experiments showed very hydrophobic biochar surfaces. The biochars were used as adsorbents for diclofenac and amoxicillin. They presented very high adsorption performances, which could be explained by the pore filling, hydrophobic surface, and π–π electron–donor–acceptor interactions between aromatic rings of both adsorbent and adsorbate. The biochar with the highest surface area (and highest uptake performance) was subjected to regeneration tests, showing that it can be reused multiple times.

## 1 Introduction

The worldwide demand for carbon-based materials such as activated carbon and biochar is rising rapidly, with a 10% increase per year due to a diverse range of applications such as water decontamination, gas separation, and energy storage systems, among others.^1^ In addition, due to high specific surface area (SSA) and well-developed porosity with different structures, carbon-based materials are of great interest for both research and industrial applications.^1−4^

Biochar is prepared by thermal decomposition of biomass under an inert atmosphere and commonly at high temperatures.^2−5^ The carbon properties can be improved by applying chemical activators, such as acids or alkaline impregnation techniques, to achieve high SSA and well-developed micro- and mesoporosity, as well as an abundance of functional groups on the biochar’s surface.^4−6^ To produce biochars with improved characteristics, research is required to obtain an optimized surface area, total pore volume, and distribution of pore size that can be later employed in suited applications such as adsorbents or electrodes.^7−9^ Research has shown that the carbon properties are heavily affected by factors such as the experimental conditions utilized in the pyrolysis step and further activation of the carbon-based material, i.e., process temperature, pyrolysis time, heating rate, and type of chemical activator and its amount.^7,9−12^

The factors listed above may act isolated or combined to influence the physicochemical characteristics of the biochars, which adds difficulties in identifying the process parameters needed for synthesizing high-quality biochars.^9−12^ To solve this problem, the design of experiments (DoE) and response surface methodology (RSM) are usually employed to evaluate the influence of the combined factors and optimize them to improve the characteristics of the desired material.^9−12^ RSM is cost-effective since it decreases the total number of experiments required to obtain the best conditions and the maximum response.^9−13^

RSM has been used by many researchers worldwide for the optimization of experimental conditions for biochar fabrication.^12,13^ dos Reis et al.^13^ utilized Norway spruce bark to produce porous biochars. The effect of three factors (pyrolysis time and temperature and ratio of activator agent) was examined on three responses (SSA, micropore and mesopore areas). It was found that the chemical activator ratio and the pyrolysis temperature were the most important variable affecting SSA values. The time of pyrolysis exerted the most remarkable influence on the micropore area, while the interaction between the chemical activator and temperature significantly affected the formation of mesopores. Abioye et al.^14^ focused on physical activation; the authors employed a DoE analysis to evaluate the effect of holding time, pyrolysis temperature, and CO_2_ flow rate over SSA and micropore volume of oil palm shell-based biochars. They found that the time for activation has a more remarkable effect on SSA and micropore volume values. The SSA values were within 291–574 m^2^ g^–1^, and the optimized conditions were found to be at 900 °C, a CO_2_ flow rate of 400 cm^3^ min^–1^, and a holding time of 40 min.

The characteristics of activated biochars are highly dependent on the selected activation method and which chemical activator is employed.^3,9,13^ Chemical activation with KOH is a very efficient process to obtain moderate biochar yields and highly developed microporous structures with high SSA values.^9^ The following steps can mainly summarize the KOH activation process: (i) KOH reacts with carbon in biomass to produce K_2_CO_3_ via redox reactions; (ii) formation of K_2_O by dehydration of K_2_CO_3_ by a carbonate reaction; and (iii) metallic potassium (K) interacts with the carbon matrix and expands the carbon lattices, which develop and widen the pores.^9,18,19^ Activation with KOH generates biochar with high oxygen content, increasing the hydrophilicity and facilitating reactions between the solid–liquid phase, i.e., charge storage and adsorption.

Pulp and paper mill sludge has been utilized as a carbon precursor to produce activated biochar.^15−17^ This industrial sector generates large amounts of sludge residues commonly used as fuel or sent to landfills, but it could have a more sustainable use.^20^ Only in Sweden, 257 kilo tonnes of dry sludge solids are generated annually by paper and pulp mills.^17^ To manage those large quantities, incineration of the sludge is often used. Nevertheless, the high water content in the sludge makes sludge management costly and can stand for up to 65% of the total operating costs at paper mills.^19^ Paper mill sludges are basically composed of organic matter (mainly cellulose fiber from wood or recycled paper) in which organic compounds are added to the paper or pulp while inorganic compounds (mainly calcium carbonate, kaolinite, and talc) are also utilized.^21,22^ The high organic content in sludge makes it very suitable for biochar preparation. Therefore, in this paper, sludge from a paper mill was utilized as a carbon precursor to produce activated biochar using chemical activation with KOH.

Therefore, the following goals were established as a result of this research: (i) employ the pulp and paper mill sludge as a precursor for producing biochars with well-developed porosity and high SSA values; (ii) optimize the sludge-based biochar production conditions by KOH chemical activation by simultaneous evaluation of the pyrolysis temperature, holding time and impregnation ratio; (iii) obtain biochars with both desirable micro- and mesoporosities; (iv) test the biochars effectiveness for the removal of two pharmaceuticals (sodium diclofenac and amoxicillin); and (v) evaluate the recyclability of the biochar through successive adsorption/desorption tests.

## 2 Materials and Methods

### 2.1 Raw Material, Chemicals, and Solutions

The bio-sludge used as a precursor was provided from the biological wastewater treatment plant at Holmen Paper AB, Sweden. KOH (pellets, ≥86%) was used as a chemical activating agent. Sodium diclofenac and amoxicillin (both of 99.99% purity) were acquired from Merck and used without any previous purification. All of the solutions used in this work were prepared using deionized water.

### 2.2 Biochar Preparation

The activation process was performed using a one-step method (simultaneous carbonization and activation).^3,9^ First, dried sludge (10.0 g) was well mixed with KOH, and about 30.0 mL of deionized water was added and mixed for 5 min to form a paste. The resulting paste was dried in an oven overnight at 105 °C. The impregnated precursor was then pyrolyzed at selected temperatures and holding times using a fixed heating rate of 10 °C min^–1^ at an inert atmosphere (100 mL min^–1^ of N_2_ flow).^3,9^ After pyrolysis, the samples were cooled down under N_2_ gas flow until they reached a temperature of 150 °C. After that, the N_2_ gas was closed, and the samples were allowed to cool down to room temperature overnight. Finally, the carbons were washed with deionized water until a neutral pH was obtained.

### 2.3 Biochar Characterization

The specific surface area and porosity of pyrolyzed sludge mill biochars were obtained using a Micromeritics 3Flex physisorption instrument (Micromeritics Instruments, Norcross, GA). Amounts between 100 and 200 mg of each biochar were degassed under vacuum at 140 °C for 3 h. 3Flex version 5.02 software was used to process the isotherm data.

The biochar morphology was evaluated by scanning electron microscopy (SEM) using a Carl Zeiss Merlin model.

Raman spectroscopy was carried out to obtain information on the bulk of carbon materials. Raman spectra were recorded on a Renishaw inVia Raman spectrometer (Renishaw, Kingswood, U.K.) at 633 nm HeNe laser in 45–4500/cm.

The hydrophobicity/hydrophilicity index (HI) was measured as previously reported.^9,13^ Based on adsorption in saturated atmospheres by two solvent vapors, water (hydrophilic) and *n*-heptane (hydrophobic), the weight gained during vapor adsorption was used to calculate the hydrophilicity/hydrophobicity index of the biochars.

### 2.4 Experimental Design

The production and optimization of the bio-sludge-based biochars were carried out according to a Box–Behnken experimental design (BBD) and a response surface methodology (RSM).^13^ The RSM is a useful statistical tool that allows simultaneously observing the effect of more than one process variable on the evaluated response(s). The BBD was applied to correlate three responses: (i) specific surface area SSA, (ii) micropore surface area, and (iii) mesopore surface area for three biochar preparation factors, (i) pyrolysis temperature, (ii) holding time, and (iii) ratio of the biomass:KOH.

The experimental design used in this work was composed of 15 experiments, including 12 factorial points and 3 center points (Table 1). Minitab software (version 20) was employed to evaluate factors’ influence on the responses.

**Table 1:** Experimental BBD Design Matrix with 15 Runs with 3 Central Points

	coded levels			encoded levels		
coded samples	temperature (°C)	holding time (h)	sludge:KOH ratio	temperature (°C)	holding time (h)	sludge:KOH ratio
SB1	–1	–1	0	700	1	1.5
SB2	1	–1	0	900	1	1.5
SB3	–1	1	0	700	3	1.5
SB4	1	1	0	900	3	1.5
SB5	–1	0	–1	700	2	1
SB6	1	0	–1	900	2	1
SB7	–1	0	1	700	2	2
SB8	1	0	1	900	2	2
SB9	0	–1	–1	800	1	1
SB10	0	1	–1	800	3	1
SB11	0	–1	1	800	1	2
SB12	0	1	1	800	3	2
SB13	0	0	0	800	2	1.5
SB14	0	0	0	800	2	1.5
SB15	0	0	0	800	2	1.5

### 2.5 Adsorption Experiments

Stock solutions for sodium diclofenac (DCF) and amoxicillin (AMX) of 1000.0 mg L^–1^ were prepared and used for the adsorption tests. First, 30 mg of each biochar were added to 20 mL of each DCF and AMX adsorbate in 50 mL Falcon tubes and agitated for 4 h. The experimental adsorption tests were carried out under the initial pH of 6.0, and at 298 K. After agitating the slurry (adsorbent + sorbing solution), the liquid phase was separated by centrifugation. The concentration of DCF and AMX in depleted solutions was quantified using a UV–vis spectrometer at a maximum wavelength of 228 and 285 nm, respectively. The adsorption capacity of the biochars for both drugs was obtained according to eq 1
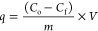
1where *m* is the adsorbent weight (g), *C*_o_ and *C*_f_ are the initial and final drug concentrations (mg L^–1^), respectively, *q* is the adsorption capacity (mg g^–1^), and *V* is the volume of the drug solution (L).

All experiments were duplicated, and blank tests were done to check for deviations.

For the desorption (regeneration) tests, DFC- and AMX-laden biochar were mixed with deionized water and subsequently shaken. This step was utilized to remove the unadsorbed pharmaceuticals and dried for 12 h at 70 °C. Next, the biochar loaded with the two drugs was immersed in 0.1 M NaOH + 20% EtOH eluent and shaken for 5 h. Then, the released DFC and AMX were separated by centrifugation from the solid adsorbent sample. Next, the solid phase was washed with deionized water to remove the remaining eluent phase, and finally, the biochar was dried again, as previously reported.^2,3^ The sorption capacity of the reutilized biochar was determined again. Four cycles of adsorption–desorption were performed (in triplicate).

## 3 Results and Discussion

### 3.1 Sludge Elementary Analysis

The results of the elemental analysis show that the sludge is mainly composed of carbon (49.3%), oxygen (30.4%), hydrogen (6.0%), nitrogen (3.2%), and 11.1% of other inorganic elements such silicon, aluminum, calcium, iron, kaolin, etc. (Table 2). Kaolin and calcium are widely used as particulate minerals in the filling and coating paper.^23^ The large amounts of carbon, oxygen, and hydrogen make the selected sludge suitable for biochar preparation. High carbon content helps develop the biochar’s bulk structure and porosity. In addition, the oxygen, hydrogen, and nitrogen content increases the number of functional groups on biochar surfaces essential for efficient biochar applications.

**Table 2:** Chemical Composition of Pulp and Paper Sludge

elements	quantity (%)
carbon	49.3
oxygen	30.4
hydrogen	6.0
nitrogen	3.2
other elements	quantity
silicon	19 000
aluminum	9100
calcium	7700
iron	4600
sodium	4000
magnesium	1300
phosphorous	3900
kaolin	1000

### 3.2 Textural Characteristics of the Sludge Biochars

The biochars’ textural properties, such as SSA, *S*_meso_, *S*_micro_, and pore volume, have an essential effect on their effectiveness in different applications. For instance, adsorption and electrochemical performance are often connected to textural properties.^2,3,9^

Table 3 shows the SSA, *S*_meso_, *S*_micro_, and pore volume of biochars made from pulp and paper bio-sludge. A notable difference was observed for the SSA values, ranging from 273 to 885 m^2^ g^–1^, depending on the preparation conditions, which indicates that the bio-sludge is a suitable precursor for highly porous biochar preparation. Table 3 also shows the *S*_micro_ and *S*_meso_ values; the sludge biochars can be predominantly micro- or mesoporous, depending on the preparation conditions. *S*_micro_ is one of the crucial characteristics of biochars because it contributes a lot to the SSA, while *S*_meso_ is very important in liquid-phase adsorption.

**Table 3:** Box–Behnken Design of Experiments and Textural Properties of the Biochars

sample ID	SSA (m^2^ g^–1^)	*S*_micro_ (m^2^ g^–1^)	*S*_meso_ (m^2^ g^–1^)
SB1	398	263	135
SB2	538	258	280
SB3	570	393	177
SB4	420	189	231
SB5	541	379	162
SB6	636	305	331
SB7	633	411	222
SB8	273	109	164
SB9	837	569	269
SB10	885	469	416
SB11	666	420	246
SB12	327	111	216
SB13	860	387	473
SB14	857	411	446
SB15	832	366	466

Table 3 shows that eight carbons presented more micropores in their structures among the fifteen biochars, while seven contained mesopores predominantly. Both types of pores are highly desirable for a wide range of applications, especially for energy storage applications and adsorbent materials.^1,7,13^

Previously dos Reis et al.^24^ produced biochars employing sewage sludge as a precursor. These experiments achieved specific surface areas up to 679 m^2^ g^–1^. Negara et al.^25^ employed tabah bamboo to produce biochars with a predominance of micropores with an SSA of 398 m^2^ g^–1^. Mistar et al.^26^ used bamboo wastes to make biochars with microporous structures, and the microporous volume was augmented with both activator agent:biomass ratio and pyrolysis temperature. Finally, Galiatsatou et al.^27^ produced biochars from olive pulp and peach stones and reported that longer holding times favored the creation of mesopores.

The above results make it difficult to correlate the SSA, *S*_meso_, and *S*_micro_ values reliably with the pyrolysis and preparation parameters. In this sense, employing a DoE makes it easier to analyze and identify which and how biochar preparation parameters influence the biochar textural properties. Therefore, the following section is dedicated to exploring the DoE on the main textural properties of the biochars (SSA, *S*_meso_, and *S*_micro_).

### 3.3 Box–Behnken Design of Experiments

A Box–Behnken design of experiments with 3 factors (pyrolysis temperature, holding time at the final temperature, and the ratio of biomass:KOH) was performed for three responses, Brunauer–Emmett–Teller (BET) surface area (SSA), micropore area (*S*_micro_), and mesopore area (*S*_meso_). Usually, the confidence interval established for Statistical Design of Experiments is 95% (probability of 5%) because most analysis surface responses show less than 5% variation.^11,14^ However, the variation coefficient for the microporous area for the central point (experiments 13–15) was 5.84%; therefore, in this current RSM, a probability of 10% was established.

**Figure 1:**
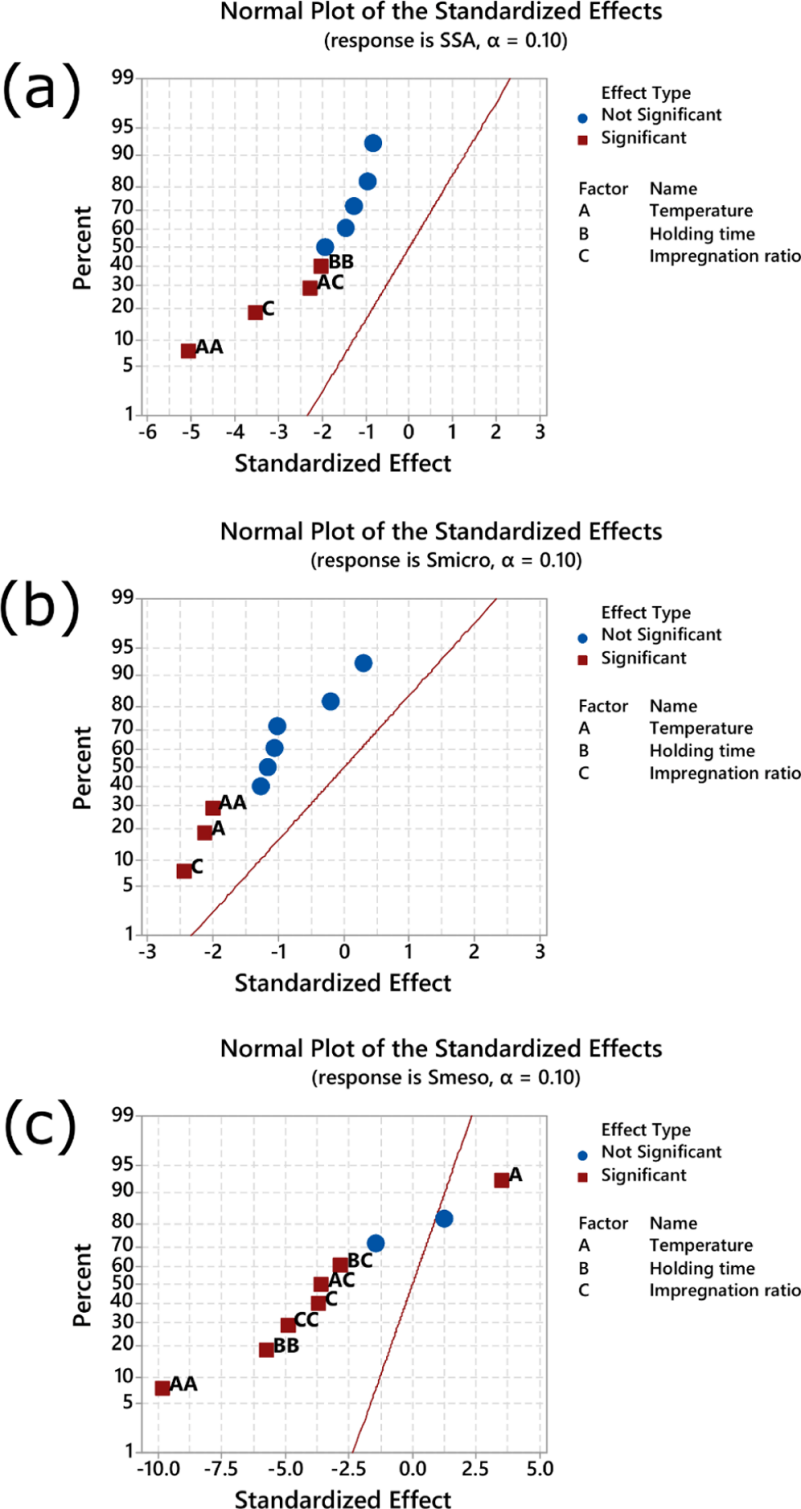
Normal plot for standardized effect for SSA (a), *S*_micro_ (b), and *S*_meso_ (c).

The analysis of variance of the responses SSA, *S*_micro_, and *S*_meso_ are presented in Supporting Tables 2–4, respectively, and the normal plot of standardized effects is shown in Figure 1 (α 0.10). The red squares display the significant effects in the plot, and the blue circles show the nonsignificant. Another critical aspect is the distribution of the points on the positive (at right) and negative side (at left) of the standardized effects. When the significant effect is to the right of the standardized, an increased factor level increases the response value and vice versa. For example, observing Figure 1, except for effect temperature (*A*) for the response *S*_meso_, all of the significant points are at the left of standardized effects. This means that the temperature positively affected the mesopore area, meaning that with the increase in temperature, the mesopore area value increased as well. However, since the other factors are located on the left side of the graph, their increases cause a decrease in their SSA, *S*_micro_, and *S*_meso_ values.

The Box–Behnken response surface methodology had a reasonable fitting for all of the responses, attaining values of *R*^2^ of 92% (SSA), 80% (*S*_micro_), and 97% (*S*_meso_). The lack of fit was significant for SSA and *S*_micro_ responses (Supporting Tables 1 and 2) but not for *S*_meso_ (Supporting Table 3).

For the response SSA, the contribution from each significant factor was 21% for the ratio biomass:activator agent (*C*), 40% for the squared temperature (*A*^2^), 6.3% for square holding time (*B*^2^), and 9% for the interaction between temperature multiplied by the ratio of activator agent (*A*.*C*). For the response *S*_micro_, the contribution from each significant factor was 18.17% for pyrolysis temperature (*A*), 24% for the ratio of biomass:KOH (*C*), and 16.5% for the squared temperature (*A*^2^). For the response, *S*_meso_, the contribution from each significant factor was 6.3% for temperature (*A*), 7.1% for ratio biomass:KOH, 43.45% for squared temperature (*A*^2^), 15.2% for squared holding time (*B*^2^), 12.6% for squared ratio (*C*^2^), 6.7% for the interaction of the two factors temperature and ratio (*AC*), and 4.2% for the interaction of holding time and ratio (*BC*).

**Figure 2:**
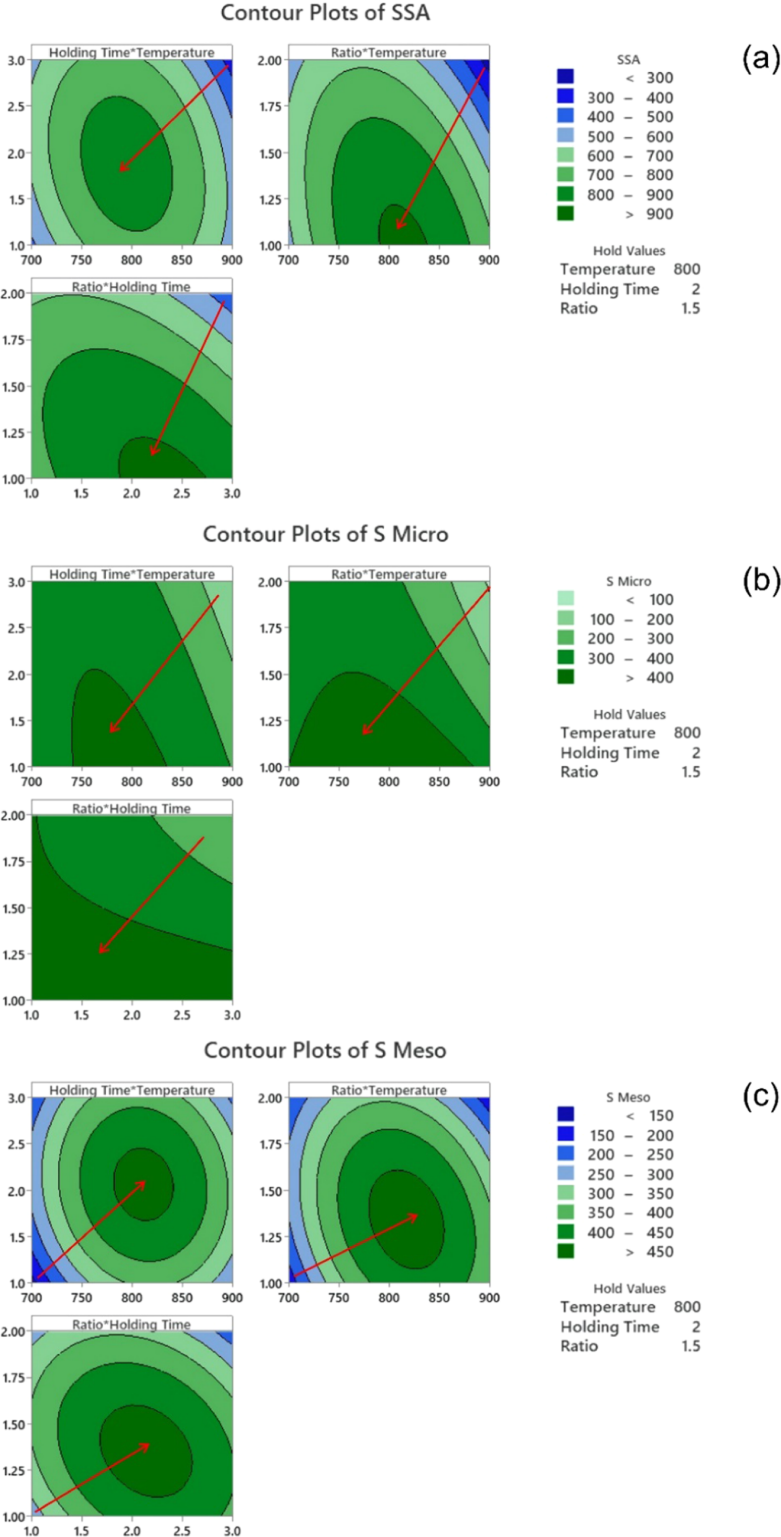
Contour plots for the effects of the pyrolysis temperature, holding time, and KOH:biomass ratio on SSA (a), *S*_micro_ (b), and *S*_meso_ (c).

Figure 2 presents contour plots for each response. The red arrows indicate the increasing values of each response. The highest SSA values (Figure 2a) occur at a pyrolysis temperature close to 800 °C, pyrolysis time of 2 h, and biomass:KOH ratio of 1:1. The highest *S*_micro_ values are obtained at 750–800 °C, 1 h, and biomass:KOH ratio of 1:1 (Figure 2b), and the highest *S*_meso_ at 800–850 °C, 2 h, and ratios of 1.25–1.50 (Figure 2c).

**Figure 3:**
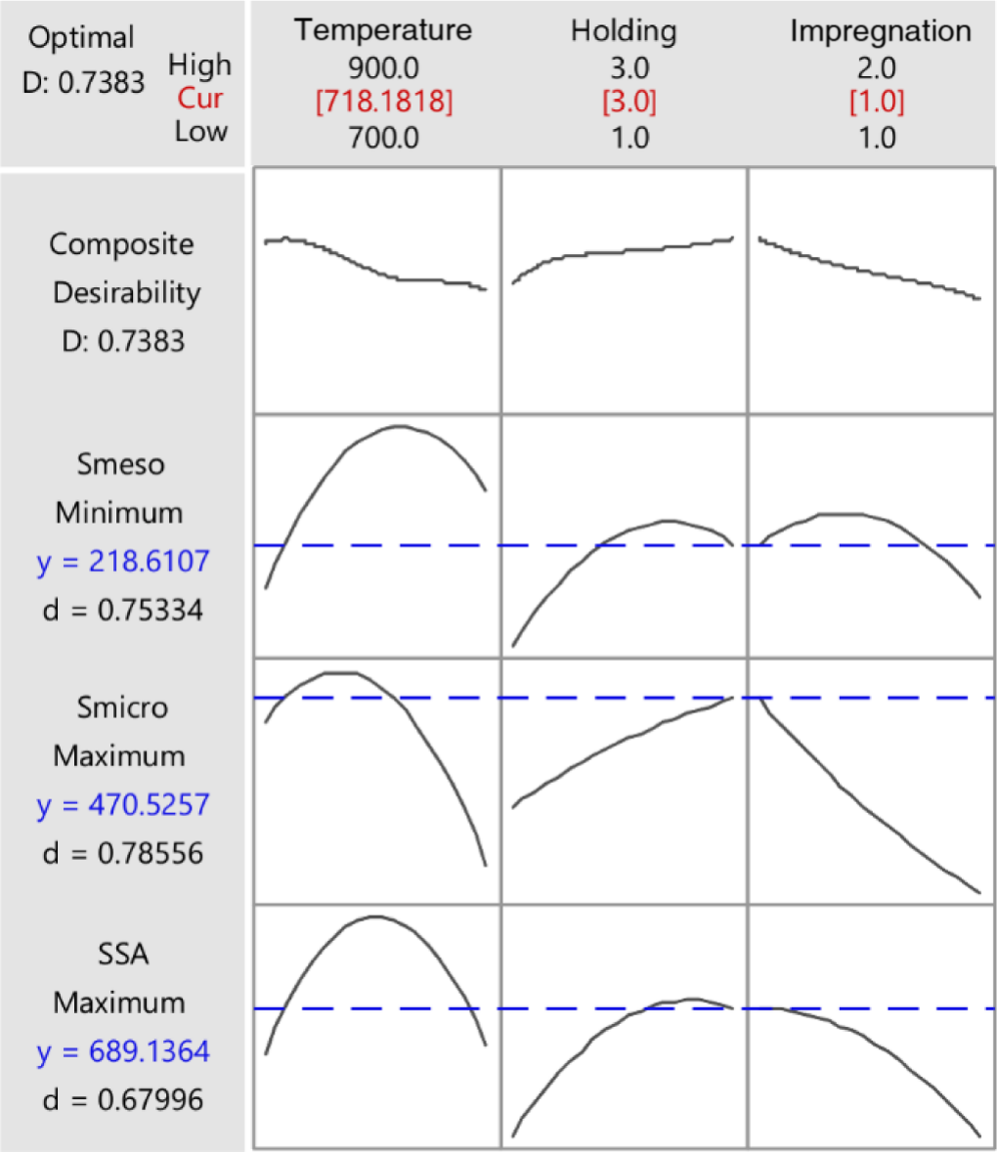
Optimization of the responses for production of sludge biochars.

The adsorbents prepared in this study aim to adsorb small molecules. Therefore, it is vital to have carbon-based materials with high surface areas, a high amount of micropores, and a lower amount of mesopores. Thus, an optimization of the three responses was made to obtain maximum SSA, maximum *S*_micro_, and minimum *S*_meso_: the ideal model conditions were a pyrolysis temperature of 718 °C, a 3 h holding time, and a ratio of biomass:KOH of 1:1, with the desirability (*D*) of 0.7383 (see Figure 3).

#### 3.3.1 Biochar Preparation: Comparison with the Literature

In Supporting Table S4, our results are compared with previous works that used DoE and RSM to prepare biochars from various carbonaceous precursors.^10,14,28−33^ Comparing results from different DoE analyses and conditions is unreliable. Still, it can be inferred that an optimum method of preparation of carbon-based materials depends on the carbon source properties and the desired outcome. Therefore, systematic understanding is required to determine the pyrolysis conditions and chemical activation, at which biochars with improved physicochemical properties are obtained. Besides, having a clear understanding of which and how the factors influence biochar properties makes it possible to adapt the pyrolysis and activation parameters to get biochar with tailored properties for target applications. Based on Supporting Table S4, it is safe to state that biochars with high SSA and microporous and mesoporous characteristics can be produced from pulp and paper mill bio-sludge, highlighting their suitability for biochar synthesis.

### 3.4 Biochar Characterization

#### 3.4.1 Field Emission Scanning Electron Microscopy (FESEM)

**Figure 4:**
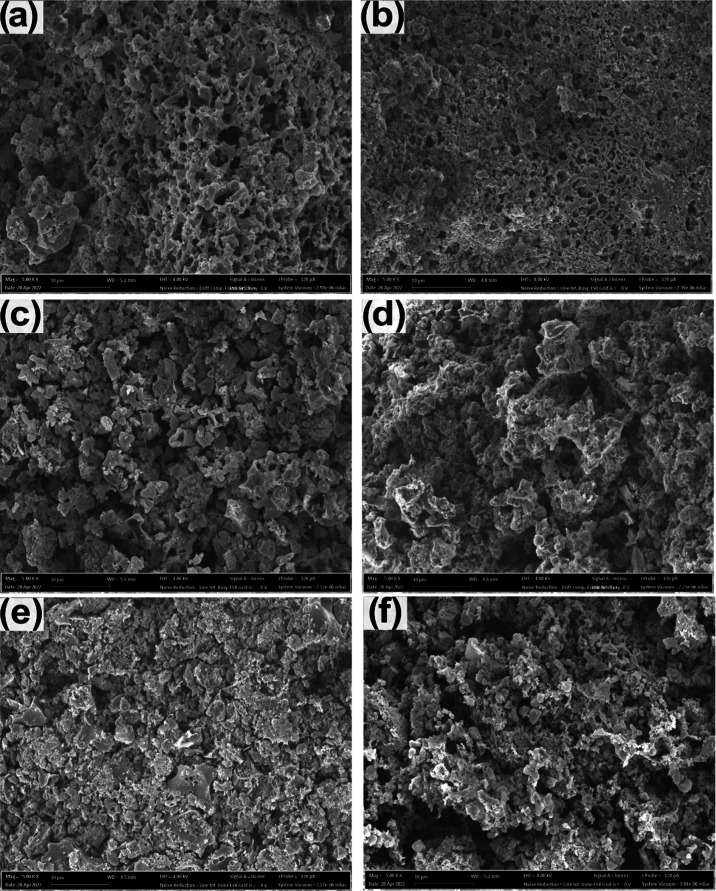
SEM images of sludge biochars samples of SB10 (a), SB13 (b), SB7 (c), SB5 (d), SB1 (e), and SB8 (f). All at 5 K of magnification.

The textural properties are well supported by the morphological analysis of biochars using field emission scanning electron microscopy (FESEM). High-quality FESEM images at magnification 5000× of biochars samples: SB13, SB10, SB7, SB5, SB1, and SB8, are shown in Figure 4.

The images highlight differences related to the pyrolysis preparation conditions. For instance, in Figure 4a, the SB10 sample has a broken, irregular structure, full of holes and cavities, and an extremely rough surface. On the other hand, sample SB13 (Figure 4b) has a less broken structure but a rough surface covered by holes. Interestingly, these two samples presented the highest SSA among all fifteen biochars. The other samples have broken structures with high roughness, but no holes are observed on their surfaces—as a consequence, they presented lower SSA than SB10 and SB13. The holes in SB10 and SB13 are macropores with considerable importance for the solid–liquid contact, allowing passage for pollutants or electrolytes to the smaller pores in the inner structure of the biochars, thereby maximizing the accumulation of pollutants (if used as adsorbent) or charge storage (if used as electrodes) into the cavities.^34,35^

#### 3.4.2 Raman Analysis

**Figure 5:**
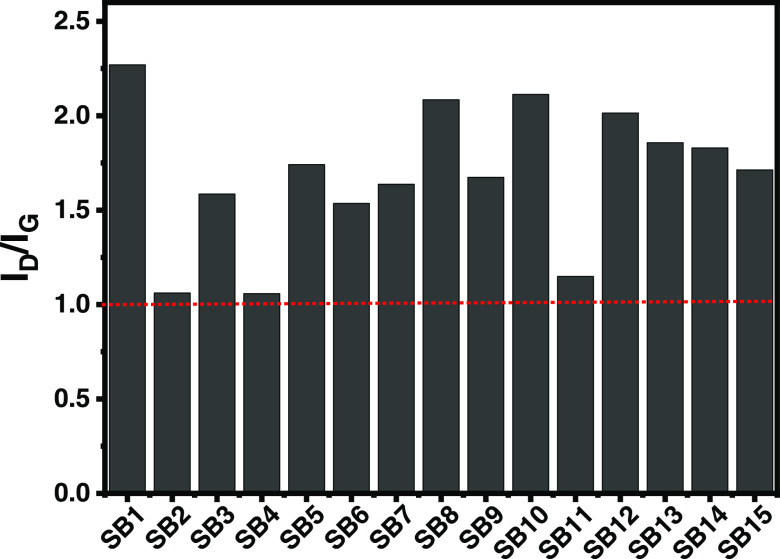
Ratio of *I*_D_/*I*_G_ bands of sludge biochars.

Raman spectroscopy analysis was performed to examine the graphitization degree of the biochars. Using Raman, it is possible to obtain an *I*_D_/*I*_G_ ratio that reveals crucial information on the degree of graphitization or graphene structure and the level of biochar’s perfection/order/disorder structures. The *I*_D_/*I*_G_ ratios of fifteen biochars are shown in Figure 5. All samples’ *I*_D_/*I*_G_ ratio is higher than 1, indicating more defects generated in the sp^2^ network during biochar formation and lower amounts of graphitic structures.

These values are different from others reported in the literature. dos Reis et al.^9^ employed spruce bark as a precursor using KOH activation and obtained an *I*_D_/*I*_G_ of 0.99, indicating a higher degree of graphitization. No clear trend in the *I*_D_/*I*_G_ values or correlation with the pyrolysis conditions is observed in this work; however, Figure 5 shows that the different preparation conditions caused important changes in the biochar structures. Interestingly, the three samples with the lowest *I*_D_/*I*_G_ values are those made at 800 and 900 °C, and the highest *I*_D_/*I*_G_ value was obtained for the sample SB1, which was made at a lower temperature. It is known that biochar graphitic structures are maximized using higher temperatures.

#### 3.4.3 XRD Analysis

**Figure 6:**
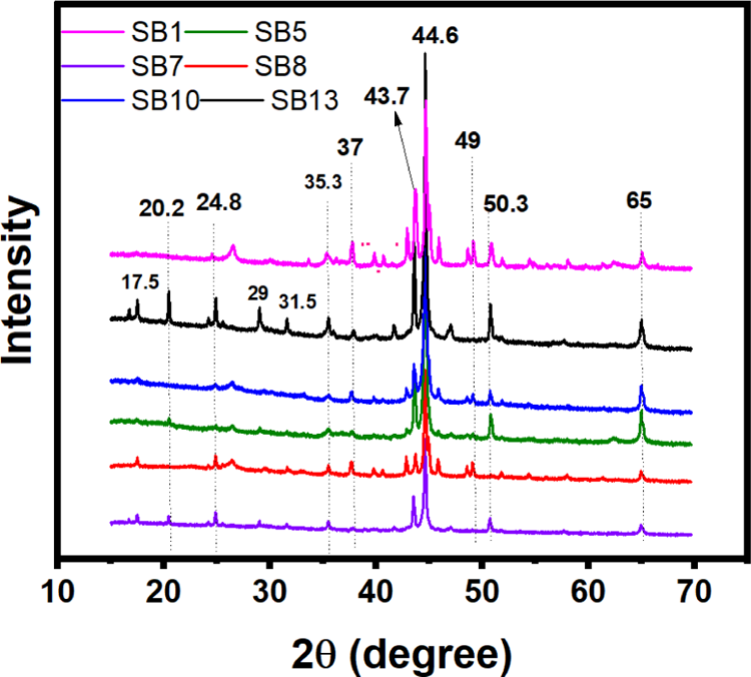
XRD patterns of sludge biochars.

X-ray diffraction (XRD) was performed to evaluate the biochar properties further (see Figure 6). The biochars present some distinct peaks pertaining to the more crystalline (impure) phase. As expected, the pyrolysis conditions affected the microstructure and the presence of crystalline phases in the biochars. The XRD peaks at 17.5. 20.2 and 24.8, 29θ suggest the presence of calcite (CaCO_3_), which is consistent with the elementary analysis that identified the presence of calcium used in paper production. The diffraction peak at 43.7θ can be related to the crystalline carbon.^36,37^ The strong diffraction peak at 44.6θ could be attributed to the quartz phase, such as SiO_2_.^36,37^ Kaolinite (Al_2_Si_2_O_5_(OH)_4_) is also identified at 50.3θ in all biochars, which matches the elementary analysis that identified Al and Si.

#### 3.4.4 Surface Characteristics

The surface properties of the prepared biochars are important for possible interactions between biochars’ surface and a selected adsorbate. Therefore, two solvents with different polarities were selected (i.e., *n*-heptane and water) to explore the surface characteristics of the biochars. If the biochar has a higher affinity for water, it presents a more polar surface and thus a more hydrophilic surface; if there is higher *n*-heptane uptake, it means that the biochar’s surface is nonpolar and hydrophobic. The *n*-heptane was chosen due to its pronounced steric factors during adsorption compared to other solvents.^13,38,39^

**Figure 7:**
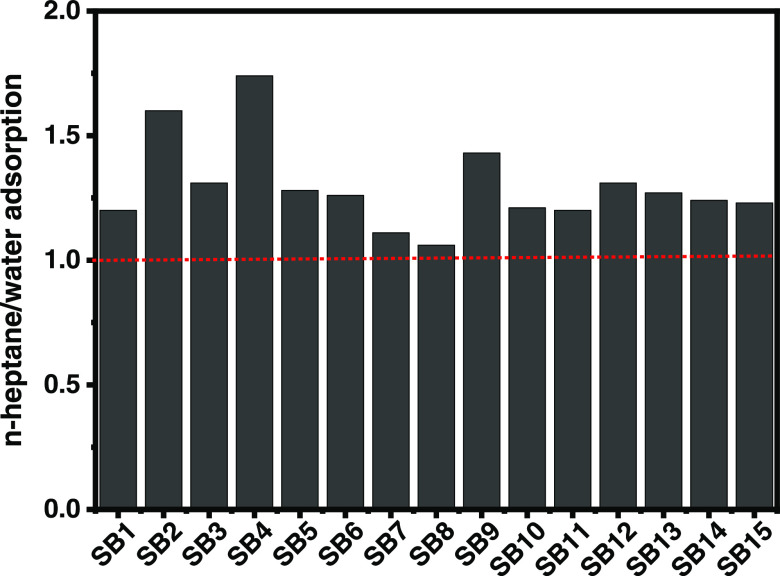
Ratio of *n*-heptane and water adsorption (hydrophobicity index values).

Hydrophobic–hydrophilic behavior of the 15 biochar samples was evaluated, and *n*-heptane:water adsorption ratios are exhibited in Figure 7. All biochars had a ratio >1, meaning a higher affinity for *n*-heptane, meaning that the surfaces were predominantly hydrophobic.^13,33,38^ Indeed, carbon-based materials are expected to be more hydrophobic than hydrophilic.^13,38,39^ However, Guy et al.^40^ prepared biochars by alkaline activation (KOH) using Norway spruce bark as a precursor and found that most biochars were hydrophilic. This contradiction could be related to the differences in the precursor; bark is more homogeneous with abundant oxygen and hydrogen groups, while paper and pulp sludge has been subjected to several extraction methods that may remove many of these hydrophilic groups, which in turn might impact the final biochar characteristics.

The hydrophobicity index of the biochars can substantially influence their performance as adsorbents because, in the adsorption process, hydrophilic and hydrophobic interactions between adsorbent and adsorbate play an essential role.^13,41^

### 3.5 Evaluation of Adsorptive Properties of Sludge Biochars against Diclofenac and Amoxicillin

**Figure 8:**
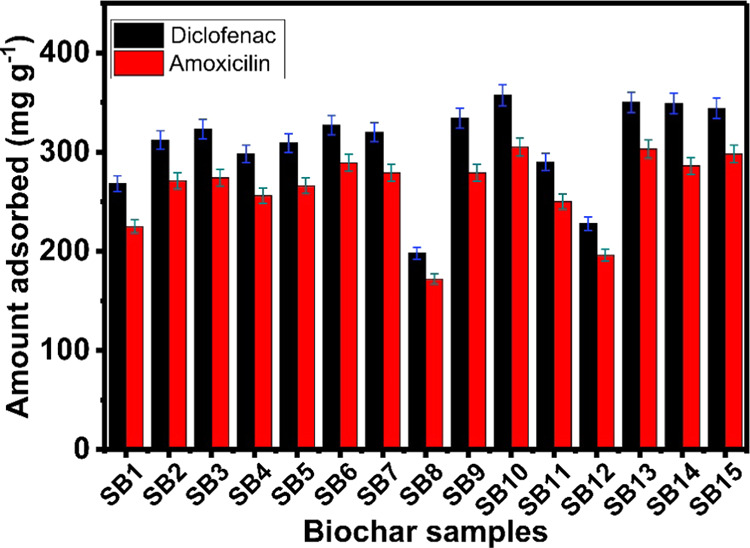
DFC and AMX adsorption capacity on pulp and paper mill sludge biochars.

The biochar materials were employed as adsorbents to remove two emerging pollutants (sodium diclofenac, DCF, and amoxicillin, AMX) from aqueous solutions. The biochar’s efficiency is based on the adsorption capacity (*q*_e_). The biochar adsorptive performances are displayed in Figure 8, showing that all biochars presented excellent adsorption capacities to remove both pharmaceuticals. Furthermore, all biochars were more effective in removing DCF than AMX, which could be related to the size of its molecule. For instance, DCF has a molecule size of 1.015 nm and AMX 1.361 nm.^42^ Since the biochars are rich in micro-and mesopores; smaller molecules can be more easily adsorbed than bigger ones due to the easier and faster diffusion process through the pores. Besides, DFC’s hydrophilic-lipophilic balance (HLB) value is 21.92, while AMX’s is 19.72 (see Supporting Figure S1). HLB is related to the compound’s hydrophilicity; a high HLB value indicates high water solubility that can improve the contact with the biochar in water to reach high adsorption values.

Despite varying textural properties, all biochars showed a high *q*_e_ for both pharmaceuticals. This performance could be justified by the beneficial pore structure and high SSA. Several works have linked the *q*_e_ with the adsorbent materials’ pore structure and SSA values.^13,42,43^ High SSA values often correlate with more active sites for interaction with adsorbates. Supporting Figure S2 shows the correlation between SSA and *q*_e_ for both adsorbates. The *R*^2^ for the SSA vs DCF removal is 0.783, while SSA vs AMX is 0.739, indicating a specific correlation between SSA and *q*_e_. However, adsorption can also depend on other properties, such as the chemical features of the biochars, surface functionalities, hydrophobicity, and adsorbate properties.

A helpful way of evaluating the effectiveness of the sludge biochars is to compare them with various adsorbent materials commonly reported in the literature; *q*_e_ is the most common parameter to assess the efficiency of an adsorbent against one or many pollutants.

Table 4 compares the adsorption capacities of various adsorbents with the sample SB10 (which presented the highest DCF and AMX removal capacities of the 15 samples). SB10 presented a very high adsorption capacity for both pharmaceuticals compared to other works, highlighting its competitiveness against even high-cost materials such graphene, carbon nanotubes, carbon xerogel, and PVA/SA/CNC@PEI (polyethyleneimine-functionalized sodium alginate/cellulose nanocrystal/poly(vinyl alcohol) core–shell microspheres). However, the preparation of these materials is highly costly and complex; even so, our low-cost paper and mill sludge biochars presented higher *q*_e_ values. Therefore, pulp and paper mill bio-sludge can be suitable for producing biochars with outstanding adsorptive properties.

**Table 4:** Comparison of the Adsorption Capacities for Diclofenac Using Different Adsorbents

adsorbent materials	molecule	*q* (mg g^1^)	pH	ref
Norway spruce bark biochar	DFC	417.4	6.0	(13)
magnetic biochar	AMX	280.9	6.0	(42)
sludge/polysiloxanes composite	DFC	26.12	7.0	(43)
reduced graphene oxide	DFC	59.67	10.0	(44)
commercial activated carbon	DFC	83	5.5	(45)
carbon nanotubes/alumina hybrid	DFC	33.9	6.0	(46)
carbon xerogel	DFC	80.0	7.0	(47)
graphene oxide nanosheets	DFC	128.7	6.2	(48)
pine tree-activated carbon	DFC	54.6	7.0	(49)
PVA/SA/CNC@PEI	DFC	444.4	5.0	(50)
magnetic graphene nanoplatelets	AMX	106.4	5.0	(51)
NH_4_Cl-induced activated carbon	AMX	437	6.0	(52)
magnetic olive kernel-activated carbon	AMX	238.1	6.0	(53)
organobentonite	AMX	196.9	7.0	(54)
chitosan	AMX	8.7	7.0	(55)
coconut shell carbon	AMX	233.7	7.0	(56)
commercial carbon	AMX	250.7	7.0	(57)
SB10	DFC	357	6.0	this work
SB10	AMX	305	6.0	this work

### 3.6 Biochar Regeneration Studies

**Figure 9:**
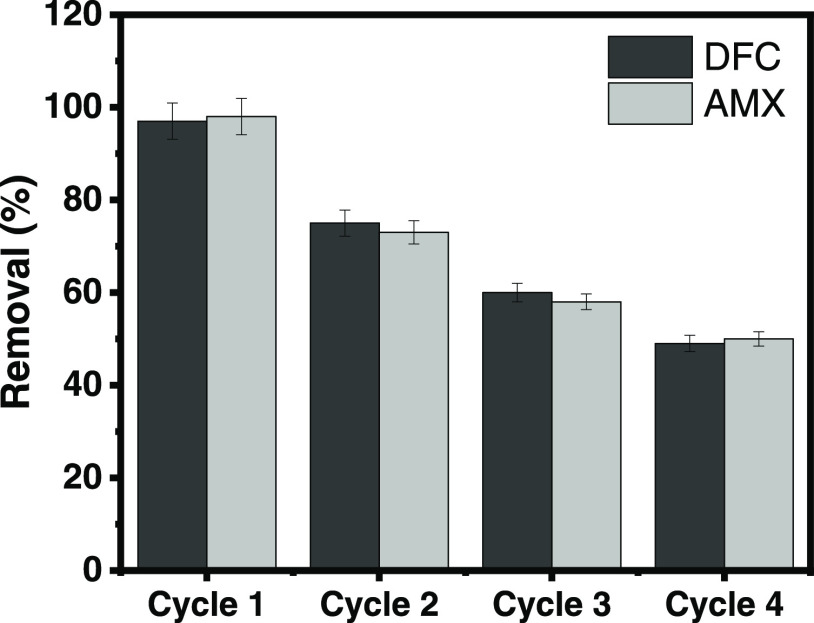
Cycles of adsorption for DFC and AMX onto SB10 using 0.1 M NaOH + 20% EtOH solution as eluent.

The regeneration step of used biochars is needed for assessing the cost-efficacy of adsorbent materials and the likelihood of future applications. The cyclability tests of SB10 using consecutive adsorption/desorption tests were performed according to the methodology described in refs (2) and (34), and the results are shown in Figure 9. Desorption tests were performed using the same procedure as the adsorption tests. 1.0 g of each drug-loaded biochar was immersed with 25 mL of NaOH + 20% EtOH solution. The flasks were stirred at 150 rpm for 1 h, and both DFC and AMX were quantified by UV–vis spectrophotometry. Four cycles of adsorption/desorption were performed to verify the recyclability of SB10. The results demonstrated that 97% of DFC and AMX were adsorbed from SB10 in the first cycle, which means that the eluent was highly effective in desorbing both drugs. In the second cycle, 75 and 73% of DFC and AMX were desorbed. After four successive cycles, nearly 51% was desorbed for both samples, suggesting that the pulp and paper mill biochar provided satisfactory recyclability performance. It can be considered sustainable and environmentally friendly as it can be employed multiple times before being considered less useful.

## 4 Conclusions

This work investigated using pulp and paper mill bio-sludge as an environmentally sustainable and low-cost precursor to synthesize porous biochars via KOH chemical activation. A Box–Behnken design was employed to target the settings for maximizing the prepared sludge biochars’ SSA, *S*_micro_, and *S*_meso_ values. Sludge biochars with SSA values up to 885 m^2^ g^–1^ were obtained. Biochars with both microporous (68% of micropores) and mesoporous (64% of micropores) features were produced and varied with different settings. Raman’s analysis indicated that all biochars presented disordered carbon structures with structural defects that can lead to improved adsorption properties. The hydrophobicity–hydrophilicity tests revealed very hydrophobic biochar surfaces. The adsorption performance compared well with the literature data when employed as adsorbents for diclofenac and amoxicillin. The biochar with the highest surface area (and highest uptake performance) was subjected to regeneration tests, showing that it can be reused several times.

The above results support the development of efficient and low-cost techniques for synthesizing biochars on a large scale with more control of desired pore characteristics. With more focus on real applications, future research in this direction might lead to more economical and sustainable water treatment technologies.

## Supporting Information Available

Analysis of variance for SSA response (Table S1); analysis of variance for *S*_micro_ response (Table S2); analysis of variance for *S*_meso_ response (Table S3); comparison of optimized settings for the production of activated carbons using different DOE methods (Table S4); physicochemical characteristics of DFC and AMX (Figure S1); and correlation between SSA and *q*_e_ values for DFC and AMX (Figure S2) (PDF)
