# Supplementary material for: Preparation and Characterization of Pulp and Paper Mill Sludge-Activated Biochars Using Alkaline Activation: A Box–Behnken Design Approach
Source: ACS Omega. 2022 Sep 2;7(36):32620–30. doi: 10.1021/acsomega.2c04290 (PMC9476204; doi:10.1021/acsomega.2c04290)
Supplement: Supplementary file 1 — ao2c04290_si_001.pdf [file ao2c04290_si_001.pdf]

Preparation and characterisation of pulp and paper mill sludge activated biochars using alkaline activation: A Box-Behnken design approach

Glaydson Simões dos Reis[1,*], Davide Bergna[2,3], Sari Tuomikoski[2], Alejandro Grimm[1], Eder Claudio Lima[4], Mikael Thyrel[1], Nils Skoglund[5], Ulla Lassi[2,3], Sylvia H. Larsson[1]

[1-] Department of Forest Biomaterials and Technology, Swedish University of Agricultural Sciences, Biomass Technology Centre, SE-901 83 Umeå

[2-] Research Unit of Sustainable Chemistry, University of Oulu, PO Box 4300, FI-90014, Oulu, Finland

[3-] Unit of Applied Chemistry, University of Jyvaskyla, Kokkola University Consortium Chydenius, Talonpojankatu 2B, FI-67100, Kokkola, Finland

[4-] Institute of Chemistry, Federal University of Rio Grande do Sul (UFRGS), Av. Bento Gonçalves 9500, Porto Alegre, RS, Brazil

[5-] Thermochemical Energy Conversion Laboratory, Department of Applied Physics and Electronics, Umeå University, SE-901 87 Umeå, Sweden

*Correspondence: glaydson.simoes.dos.reis@slu.se

Table S1. Analysis of Variance for SSA response.

	DF	Seq SS	Contribution	Adj SS	Adj MS	F-Falue	P-value
Model	9	539827	91.57%	539827	59981	6.03	0.031
Linear	3	141474	24.00%	141474	47158	4.74	0.063
Temperature	1	9453	1.60%	9453	9453	0.95	0.374
Holding Time	1	7021	1.19%	7021	7021	0.71	0.439
Ratio	1	125000	21.20%	125000	125000	12.58	0.016
Square	3	288129	48.87%	288129	96043	9.66	0.016
Temperature*Temperature	1	234970	39.86%	255555	255555	25.71	0.004
Holding Time*Holding Time	1	37157	6.30%	40772	40772	4.10	0.099
Ratio*Ratio	1	16003	2.71%	16003	16003	1.61	0.260
2-Way Interaction	3	110224	18.70%	110224	36741	3.70	0.097
Temperature*Holding Time	1	21025	3.57%	21025	21025	2.12	0.206
Temperature*Ratio	1	51756	8.78%	51756	51756	5.21	0.071
Holding Time*Ratio	1	37442	6.35%	37442	37442	3.77	0.110
Error	5	49699	8.43%	49699	9940		
Lack-of-Fit	3	49227	8.35%	49227	16409	69.43	0.014
Pure Error	2	473	0.08%	473	236		
Total	14	589526	100.00%				

R^2	R^2_{adj}
91.57%	76.39%

Table S2. Analysis of Variance for S_{Micro} response.

	DF	Seq SS	Contribution	Adj SS	Adj MS	F-Value	P-Value
Model	9	188081	80.04%	188081	20897.9	2.23	0.196
Linear	3	113998	48.51%	113998	37999.2	4.05	0.083
Temperature	1	42692	18.17%	42692	42691.9	4.55	0.086
Holding Time	1	15062	6.41%	15062	15062.4	1.61	0.261
Ratio	1	56243	23.93%	56243	56243.2	5.99	0.058
Square	3	40160	17.09%	40160	13386.8	1.43	0.339
Temperature*Temperature	1	38846	16.53%	38199	38199.1	4.07	0.100
Holding Time*Holding Time	1	514	0.22%	417	417.1	0.04	0.841
Ratio*Ratio	1	800	0.34%	800	800.5	0.09	0.782
2-Way Interaction	3	33923	14.44%	33923	11307.7	1.21	0.398
Temperature*Holding Time	1	9986	4.25%	9986	9986.0	1.06	0.350
Temperature*Ratio	1	13091	5.57%	13091	13090.8	1.40	0.291
Holding Time*Ratio	1	10846	4.62%	10846	10846.2	1.16	0.331
Error	5	46912	19.96%	46912	9382.4		
Lack-of-Fit	3	45885	19.53%	45885	15295.0	29.78	0.033
Pure Error	2	1027	0.44%	1027	513.6		
Total	14	234993	100.00%				

R^2	R^2_{adj}
80.04%	44.10%

Table S3. Analysis of Variance for S_{Meso} response.

	DF	Seq SS	Contribution	Adj SS	Adj MS	F-Value	P-Value
Model	9	185467	97.40%	185467	20607.4	20.84	0.002
Linear	3	27031	14.20%	27031	9010.4	9.11	0.018
Temperature	1	11967	6.28%	11967	11966.8	12.10	0.018
Holding Time	1	1516	0.80%	1516	1516.1	1.53	0.271
Ratio	1	13548	7.12%	13548	13548.2	13.70	0.014
Square	3	135632	71.23%	135632	45210.7	45.73	0.000
Temperature*Temperature	1	82738	43.45%	96149	96148.7	97.24	0.000
Holding Time*Holding Time	1	28933	15.19%	32942	32941.8	33.32	0.002
Ratio*Ratio	1	23961	12.58%	23961	23961.3	24.23	0.004
2-Way Interaction	3	22804	11.98%	22804	7601.3	7.69	0.025
Temperature*Holding Time	1	2031	1.07%	2031	2031.3	2.05	0.211
Temperature*Ratio	1	12788	6.72%	12788	12788.2	12.93	0.016
Holding Time*Ratio	1	7984	4.19%	7984	7984.3	8.08	0.036
Error	5	4944	2.60%	4944	988.7		
Lack-of-Fit	3	4540	2.38%	4540	1513.4	7.50	0.120
Pure Error	2	403	0.21%	403	201.7		
Total	14	190411	100.00%				

R^2	R^2_{adj}
97.40%	92.73%

Table **S4.** Comparison of optimised settings for the production of activated carbons using different DOE methods

**Carbon source**	**Statistical methodology**	**Activator agent**	**Factors and levels**	**responses**	**Main factor**	**Principal outcomes**	**Ref.**
Banana trunk	Rotatable central composite N=15	H_3PO_4	Activation time: 35.5–134.5 min<br>Activation temperature: 367–932 ◦C<br>H_3PO_4 concentrations: 0.36–8.14 mol/L	SSA, S_{micro}, S_{meso}	Pyrolysis temperature and H_3PO_4 showed to be the most significant for SSA: S_{micro} was strongly affected by		[10]
Coconut shell	Central composite design N= 20	KOH	Temperature: 397.73-902.27 ◦C<br>Holding Time: 2 - 150 min<br>Chemical ratio: 1:1 - 3.68:1	SSA	SSA was most affected by pyrolysis temperature	SSA 343 to 1307 $m^2 g^{-1}$<br>Optimum preparation condition at 902 °C, 54.3 min, and the ratio of 3.6	[28]
Oil palm shell	Box–Behnken design N= 15	CO_2	Temperature: 800 - 900 ◦C<br>Holding Time: 20 - 40 min<br>CO_2 flow rate: 200 - 400 $cm^3 min^{-1}$	SSA and S_{micro}	Holding time was most influential on SSA and micropore volume	SSA values from 291 to 574 $m^2 g^{-1}$<br>Optimum preparation condition at 900 ◦C, 40 min and CO_2 flow of 400 $cm^3 min^{-1}$	[14]

Sludge sewage	Central composite design (face centre cube) N=15	$ZnCl_2$ and H_2SO_4	Temperature: 300 - 600 ◦C<br>Holding Time: 30 - 90 min<br>Chemical ratio: 1 – 5M	SSA and S_{micro}	The pyrolysis temperature had the greatest impact on SSA	SSA ranges from 187.5 to 307 m^2 g^{-1} for $ZnCl_2$ and from184.3 to 297.1 307 m^2 g^{-1} for H_2SO_4<br>Optimal conditions were 600 ◦C, 31 min and 5 M for $ZnCl_2$ activation and 464 ◦C, 58 min and 5 M for H_2SO_4 activation.[26]	[30]
Waste polyester textiles	Box–Behnken design N=15	$MgCl_2$	Temperature: 600 - 900 ◦C<br>Holding Time: 30 - 90 min<br>Chemical ratio: 2:1 - 12:1	SSA	SSA was affected by pyrolysis temperature, among the other factors	SSA values from 486 to 1527 m^2 g^{-1}<br>Optimum preparation condition at 900 ◦C, 90 min and the ratio of 5:1	[29]
Sewage sludge	Box-Behnken Design N= 17	KOH	Temperature: 500 - 800 ◦C<br>Holding Time: 60 - 140 min<br>Chemical ratio: 0.5:1 - 1.5:1	SSA	Holding time was the most significant factor, followed by KOH ratio and pyrolysis temperature	SSA values between 23.4 and 372.7 m^2 g^{-1}<br>Optimum preparation condition at 500 ℃, 3h and ratio of 1:1	[31]

Cassava stem	D-optimal design N= 12	none	Temperature: 450 - 900 ◦C Holding Time: 20 - 150 min	SSA	SSA was most affected by pyrolysis temperature	SSA up to 596.73 $m^2 g^{-1}$ The optimum preparation condition at 787 ◦C for 146 min	[33]
Olive-waste cakes	Doehlert matrix	none	Temperature: 750 - 850 ◦C Holding Time: 30 - 70 min	SSA	SSA was most affected by pyrolysis temperature followed by holding time	SSA values between 514 and 1271 $m^2 g^{-1}$ Optimum preparation condition at 900 °C, 90 min and 5:1	[32]

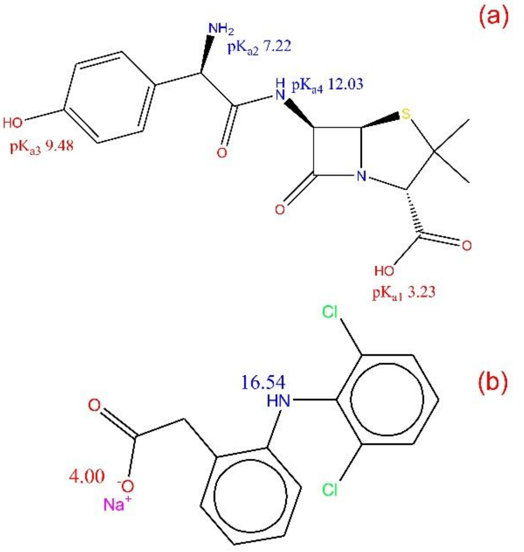

Figure S1A. (a) Structural formula of amoxicillin, pKa values are indicated in the figure; (b) Optimized three-dimensional structural formula of amoxicillin. The dimensions of the chemical molecule was calculated using MarvinSketch version 16.6.6.0. van der Waals surface area 476.14 A^2 (pH 7.0); Polar surface area 162.71 A^2 (pH 7.0); Dipole Moment 5.25 Debye; LogP -0.04; Log D -2.31. Hydrophilic-lipophilic balance 19.72

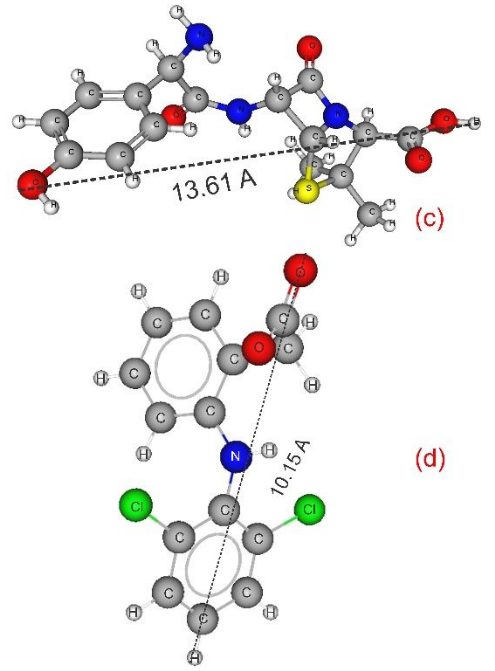

Figure S1B: (c) Structural formula of DCF; (d) Optimized three-dimensional structural formula of DCF. The dimensions and physical-chemical properties of the chemical molecule was calculated using MarvinSketch version 16.6.6. Values of pK_a are given close to the atom. Hydrophilic-lipophilic balance (HLB) 21.92; Van der Waals surface area = 359.64 A^2 (pH 4.0-14.0); Polar surface area 52.16 A^2 (pH 4.0-14.0); Dipole Moment 19.21 Debye, Log P 4.26 (non ionic); Log P 0.73 (ionic).

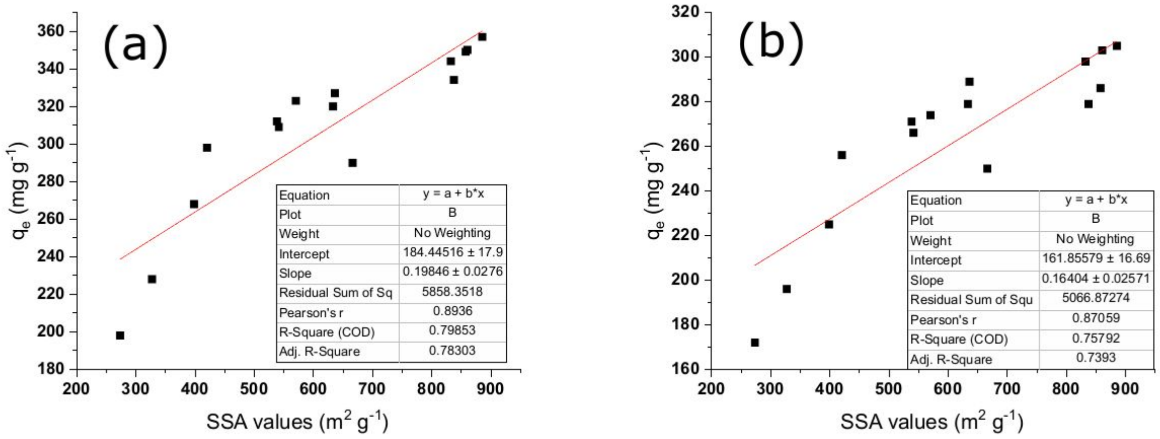

Figure S2 - Correlation between SSA and qe values for DFC (a) and AMX (b)
